# *Streptococcus suis*-associated neonatal meningitis and sepsis: characterization, antimicrobial resistance, and public health implications

**DOI:** 10.3389/fmicb.2025.1519247

**Published:** 2025-01-28

**Authors:** Giovanna Fusco, Rubina Paradiso, Lorena Cardillo, Maria Antonia Salvia, Saveria Dodaro, Veronica Del Monaco, Gianfranco Scarpelli, Francesca Greco, Antonio Rinaldi, Lorella Barca, Stefania Ambrogio, Antonio Limone, Esterina De Carlo, Giorgia Borriello

**Affiliations:** ^1^Department of Animal Health, Istituto Zooprofilattico Sperimentale del Mezzogiorno, Naples, Italy; ^2^Departmental Unit of Genetics, Bioinformatics, and Biobank, Istituto Zooprofilattico Sperimentale del Mezzogiorno, Naples, Italy; ^3^Unit of Neonatology and Neonatal Intensive Care, “Annunziata” Hub Hospital, Azienda Ospedaliera di Cosenza, Cosenza, Italy; ^4^Unit of Microbiology and Virology, “Annunziata” Hub Hospital, Azienda Ospedaliera di Cosenza, Cosenza, Italy; ^5^Cosenza Section, Istituto Zooprofilattico Sperimentale del Mezzogiorno, Cosenza, Italy; ^6^General Direction, Istituto Zooprofilattico Sperimentale del Mezzogiorno, Naples, Italy; ^7^Sanitary Direction, Istituto Zooprofilattico Sperimentale del Mezzogiorno, Naples, Italy

**Keywords:** *Streptococcus suis*, neonatal meningitis, molecular characterization, antimicrobial resistance, vertical transmission

## Abstract

Neonatal meningitis and sepsis were diagnosed in a 32-day-old preterm-born infant. *Streptococcus (S.) suis* was isolated from cerebrospinal fluid and blood. Next-generation sequencing revealed that the strain was serotype 2 sequence type 1, and contained the *ermB* and *tet(W)* genes, which are responsible for resistance to macrolides and tetracycline, along with several pilus-associated genes and 20 virulence factors. High homology was observed with previously identified human and swine strains in the same area. *S. suis* meningitis and sepsis are mainly reported in adults, related to direct contact with pigs or contaminated pork meat consumption, while it is rarely reported in children. Herein, we describe the first case of *S. suis* in a newborn associated with meningitis and antimicrobial resistance. The rates of resistance to tetracyclines, lincosamides, and macrolides for this bacterium are increasing and are creating concern worldwide. Altogether, our findings highlight the importance of investigating *S. suis* in cases of neonatal meningitis, as well as the necessity of assessing the antimicrobial profile to obtain useful information for developing targeted therapies.

## 1 Introduction

Bacterial meningitis poses a life-threatening risk to individuals of all age groups, especially infants and children in both developing and developed countries. Children under 5 years of age are at risk of meningitis followed by sepsis, which can lead to neurological disabilities and even fatal outcomes, with mortality rates reaching 100% in the absence of adequate treatment (Tavares et al., 2022). Among the most common agents responsible for bacterial meningitis are *Streptococcus* (*S*.) *pneumoniae*, *Neisseria meningitides*, *Haemophilus influenzae*, *Listeria monocytogenes*, *Escherichia coli*, *Klebsiella pneumoniae* (Wall et al., 2021). Group B *Streptococcus* has been proven to represent the main causative agent of meningitis among infants younger than 90 days (Tavares et al., 2022). Moreover, *S. suis* has acquired growing importance in the last 45 years. Indeed, while in the past it was considered a sporadic agent, the number of cases has significantly increased, with Southeast Asia being the most affected geographical area, but it has also been reported in America, Australia, and Europe (Tram et al., 2021). *S. suis* is a Gram-positive, zoonotic pathogen with a coccus shape, including 35 serotypes. However, serotypes 20, 22, 26, 32, 33, and 34 do not belong to *S. suis* (Xia et al., 2024), while serotypes 2 and 14 are the most frequently isolated from human cases. Nevertheless, it is estimated that 37% of the published cases in Europe, and 23% at the global level, are not fully characterized (Prüfer et al., 2019).

*S. suis* is a commensal bacterium that colonizes the upper respiratory tract of pigs, as well as the genital and digestive tracts. However, through the expression of toxins and degradative enzymes, it can disseminate, resulting primarily in meningitis and septicemia, as well as pneumonia, endocarditis, and arthritis (Uruén et al., 2022). The infection in humans is mainly described in adults, and it is related to the consumption of contaminated raw swine meat, but it also represents an occupational disease, primarily affecting people working with pigs (Tram et al., 2021). Nevertheless, only a few cases of *S. suis*-associated meningitis in children have been reported globally. These include a case of *S. suis* serotype 24 meningitis with a fatal outcome in 2015 in Thailand, involving a 2-year-old girl suffering from Down syndrome, and three children aged 5 to 14 in Togo (Kerdsin et al., 2016; Tall et al., 2016). To the best of our knowledge, this is the first report of *S. suis*-related neonatal meningitis and sepsis in an infant under 3 months of age.

## 2 Materials and methods

### 2.1 Sampling and bacteriological examination

In August 2023, a 32-day-old newborn was admitted to the “Annunziata” hub hospital of Cosenza, Calabria region (southern Italy), due to symptoms consistent with meningitis. Blood and cerebrospinal fluid samples were collected to define the diagnosis. Cerebrospinal fluid (CSF) was submitted to microscopic examination and a meningoencephalitis molecular panel that included 14 pathogen targets, 6 bacteria (*Escherichia coli* K1, *Haemophilus influenza*, *Listeria monocytogenes*, *Neisseria meningitides*, *Streptococcus agalactiae*, *Streptococcus pneumonia*), 7 viruses (*Cytomegalovirus*, *Enterovirus*, Herpes simplex virus 1, 2, and 6, Human parechovirus, Varicella zoster virus), and 1 yeast (*Cryptococcus neoformans*/*gattii*) (FILMARRAY Meningitis/Encephalitis, bioMérieux, Lyon, France). Furthermore, CSF and blood samples underwent bacteriological examination using blood agar (Oxoid, Rodano, Italy) and were incubated at 37°C for 24–48 h in a 5% CO2 atmosphere. After 24 h, small, slightly mucous alpha-hemolytic colonies were detected, which were identified as *Streptococcus suis* by mass spectrometry (MALDI-TOF, bioMérieux) and VITEK 2 ID & AST Cards (bioMérieux) following the manufacturer’s instructions, thus obtaining microbial identification and susceptibility testing. The results of the susceptibility test were interpreted according to the European Committee on Antimicrobial Susceptibility Testing (EUCAST). Pure colonies were also provided to the Laboratory of Genetics, Genomics, and Biobank of the Istituto Zooprofilattico del Mezzogiorno (IZSM), Portici, Naples (southern Italy) for molecular characterization.

Furthermore, after the identification of *Streptococcus suis*, nasal swabs were administered to the family components of the newborn, and a questionnaire was provided to identify the possible source of the infection.

### 2.2 DNA extraction and molecular analyses

Nasal swabs and typical colonies obtained from overnight incubation on Columbia blood agar (Oxoid) were collected for DNA extraction using a DNeasy PowerSoil kit (Qiagen, Hilde, Germany) according to the manufacturer’s instructions and then quantified using a Qubit fluorometer (Thermo Fisher Scientific, Waltham, MA, United States).

The whole-genome sequence data of the strain were generated with the Ion Torrent S5 next-generation sequencing (NGS) platform (Thermo Fisher Scientific). A total of 150 ng of bacterial DNA was used to generate a 400 bp single-end read using the Ion Xpress Fragment Library kit (Life Technologies, Carlsbad, CA, United States), loaded onto an Ion 530 chip using the Ion Chef system, and subsequently sequenced on the Ion S5 NGS platform according to the manufacturer’s instructions.

Nasal swabs were also analyzed to assess the presence of *S. suis* using a species-specific real-time PCR protocol for the detection of *Streptococcus suis* from clinical specimens (Srinivasan et al., 2016), targeting the fbpS gene. The reaction mixture was prepared in a final volume of 25 μL, including 1 μM of each primer (For-5′-TCCRATRCTGCTCTGCCATT-3′; Rev-5′-TGATAGTAGAAGTCCAGCARACT-3′), 0.5 μM probe (5′-FAM-AATAGCCC”T”GAAAAMCAGCCACWYTTTGARA-3′-6SpC; “T” = BHQ1), TaqMan Universal PCR Master Mix 1X (Applied Biosystems, Waltham, MA, United States) and 5 μL of DNA template. Thermal cycling conditions consisted of an initial denaturation step at 95°C for 10 min, followed by 40 cycles at 94°C for 30 s and 60°C for 1 min. Real-time PCR was carried out on a CFX 96 thermal cycler (Bio-Rad Laboratories, Hercules, CA, United States).

### 2.3 Data analysis

Reads produced by sequencing were checked using FastQC^1^ and *de novo* assembly was performed using the AssemblerSPAdes plugin included in the Torrent Suite^™^ Software (v. 5.18). Species identification was performed using Kraken 2,^2^ while the MLST database^3^ and Serotype tool^4^ were used to assign sequence type and serotype (Sheppard et al., 2016). Antimicrobial resistance and virulence genes were identified through the alignment of contigs against the ResFinder database^5^ and Virulence Factors Database (VFDB, http://www.mgc.ac.cn/VFs/), while pilus-associated genes were detected using BLASTN tool (v. 2.9.0+).

Finally, Snippy-core (Snippy v. 4.6) with strain 65555 (CP142676.1) as reference genome and IQtree (v. 1.6.12) with bootstrap (-b 1000) was used to infer a phylogenetic tree, including other ST1 virulent SsRC-1 (GCA_003028635.1), BM407 (GCA_000026745.1), S735 (GCA_000294495.1), GZ1 (CP000837.1), and P1/7 (AM946016.1) and avirulent LP081102 (GCA_013371025.1), hb1017 (GCA_013371575.1) strains.

## 3 Results

In August 2023, a 32-day-old preterm newborn was admitted to the emergency room of the Annunziata hospital in Cosenza, Calabria region (southern Italy), with poor reactivity to meals, nosebleeds, gasping, hypothermia, and severe clinical signs of meningitis and septic shock. The patient was intubated due to severe respiratory failure, and oxygen therapy was administered using synchronized intermittent positive pressure ventilation (SIPPV). Intravenous vasoactive amines, saline, and bicarbonates were administered due to metabolic acidosis, corticosteroids, and human immunoglobulin G. Blood and cerebrospinal fluids were collected and subjected to bacteriological analyses.

The cerebrospinal fluid examination revealed: 4,475 white blood cells/mm^3^ (74% neutrophils, 26% lymphocytes), IgG = 20.4 mg/dL (normal range: 0.00–3.40 mg/dL) albumin = 365 mg/dL (normal range: 0.00–35 mg/dL), and glucose = 1 mg/dL (normal range: 40–70 mg/dL). The meningoencephalitis molecular panel tested negative. The blood and CSF bacteriological examinations revealed the growth of small, slightly mucous alpha-hemolytic colonies on blood agar. Microscopic examination revealed the presence of Gram-positive cocci, catalase-and oxidase-negative, which were identified by mass spectrometry (MALDI-TOF), and by VITEK 2 system, as *S. suis*. The strain was also submitted to a susceptibility test, showing resistance to clindamycin. Table 1 reports the results of the susceptibility test. The patient was admitted to intensive care and underwent therapy with a full dose of sulbactam/ampicillin, ceftriaxone, gentamicin and vancomycin, omeprazole, immunoglobulin G, vasoactive amines, paracetamol, lactoferrin, lactic ferments, vitamin D, folic acid, and iron for 11 days.After a severe clinical course, the newborn showed general improvement, eupnoeic breathing, valid and rhythmic cardiac activity, improved reactivity and muscle tone.

**Table 1:** Antimicrobial resistance phenotype of *S. suis* isolates from CRF and blood matrices.

Antibiogram test	SIR	MIC	Breakpoint MIC
Benzylpenicillin	S	≤0.06	
Ampicillin	S	≤0.25	0.5
Ceftriaxone	S	≤0.12	0.5
Cefotaxime	S	≤0.12	
Clindamycin	R	> 0.5	0.5
Vancomycin	S	≤0.12	2
Teicoplanin	S	≤0.12	2

SIR interpretation according to the European Committee on Antimicrobial Susceptibility Testing—EUCAST. S, susceptible, standard dosing regimen; I, susceptible, increased exposure; R, resistant; MIC, minimum inhibitory concentration.

**Figure 1:**
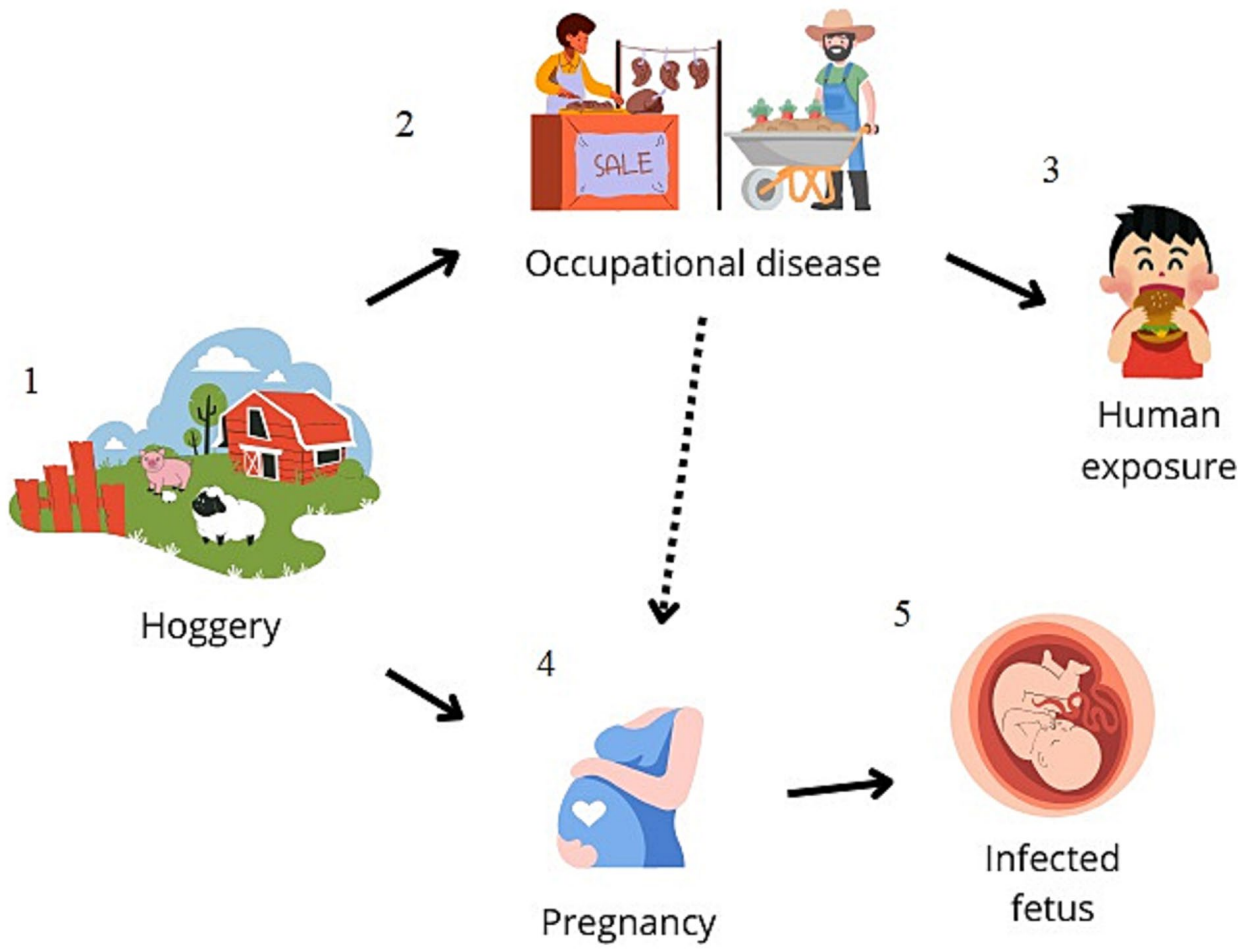
Schematic representation of the possible zoonotic route of transmission. *Streptococcus suis*-related meningitis cases are mainly reported in adults, and pig-related occupation (hoggeries) (1–2) is described to be the main risk factor for human contagion or the consumption of contaminated raw pork meat (3). In the present study, it is conceivable that the consumption of a traditional pork-based food led to the colonization of the mother (4), leading to vertical transmission to the newborn (5).

The questionnaire provided to the parents indicated that no family member was involved in activities considered at risk for transmission of *S. suis*, such as pig farming or pork meat processing. However, it was revealed that the mother regularly consumed pork sausages and had eaten a typical pork product known as “Nduja.” Furthermore, all family members tested positive by nasal swab through real-time PCR, despite the absence of clinical symptoms (Figure 1 reports a schematic of the possible route of infection).

**Figure 2:**
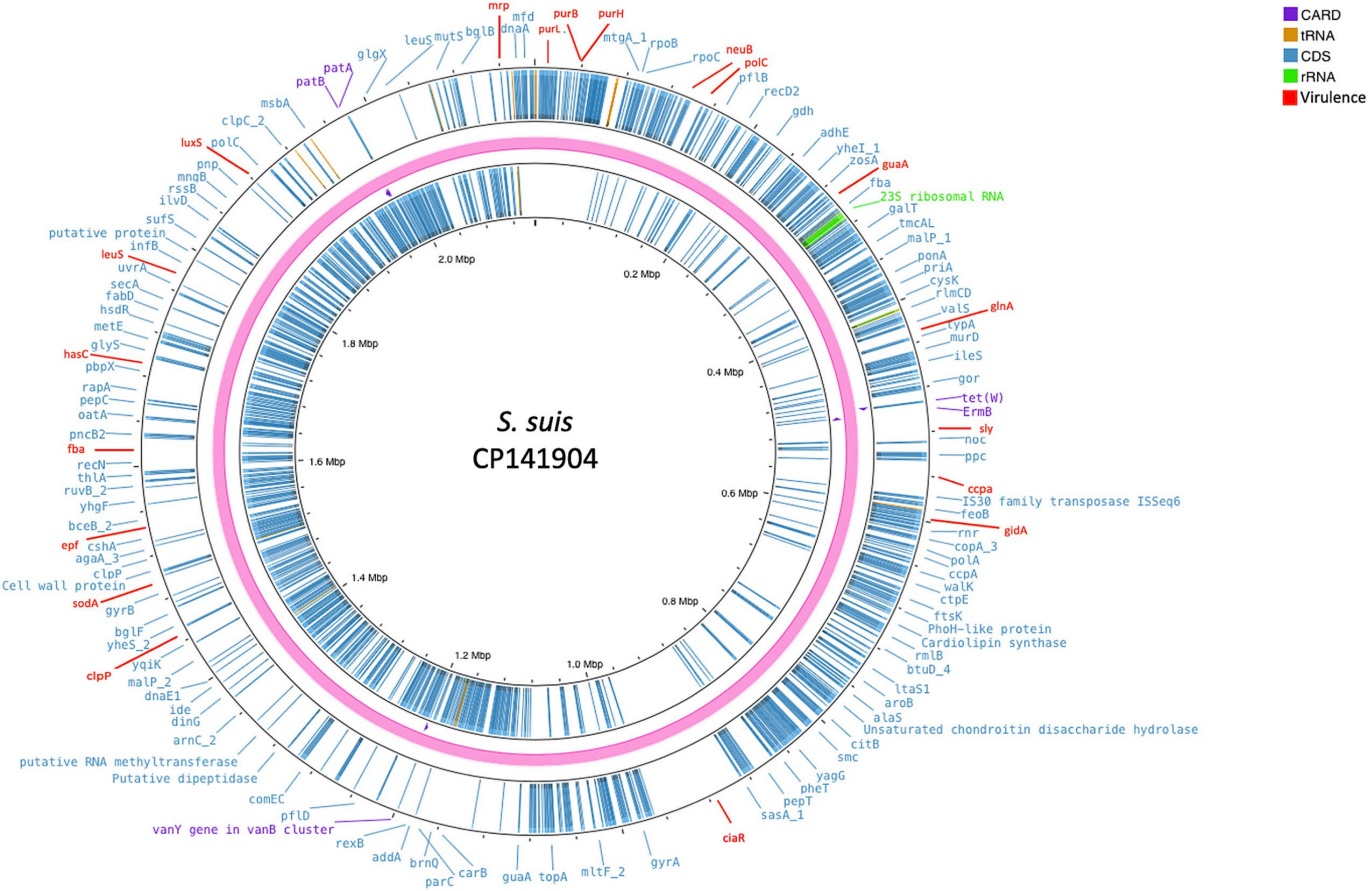
Circular chromosomal view of the *S. suis* strain under study. The outermost circle and the second circle show the position of putative protein-coding genes. The figure shows, also, the antimicrobial genes (violet), the 23S rRNA (green), virulence genes (red), and the other functional genes (blue) annotated with Prokka.

Whole-genome sequencing gave rise to 1,877,788 reads, assembled in 48 contigs, with a total length of 2,021,437 bp that were used for further analyses (Figure 2). The molecular characterization identified the strain as *S. suis* serotype 2, ST1, and revealed two genes associated with antimicrobial resistance, *ermB* and *tet(W)*, responsible for resistance to macrolides and tetracycline, respectively. Moreover, several pilus-associated genes were found related to genotype A, which are considered typical of ST1 virulent strains, including the *srtA* gene, *srtBCD* and *srtF* clusters, and *srtE* and *sipE* belonging to the *srtE* cluster.

In addition, a total of 20 genes (*ccpA*, *ciaR*, *clpP*, *epf*, *fba*, *gidA*, *glnA*, *guaA*, *hasC*, *lepA*, *leuS*, *luxS*, *mrp*, *neuB*, *polC*, *purB*, *purH*, *purL*, *sly*, and *sodA*) and virulence-related factors were observed.

**Figure 3:**
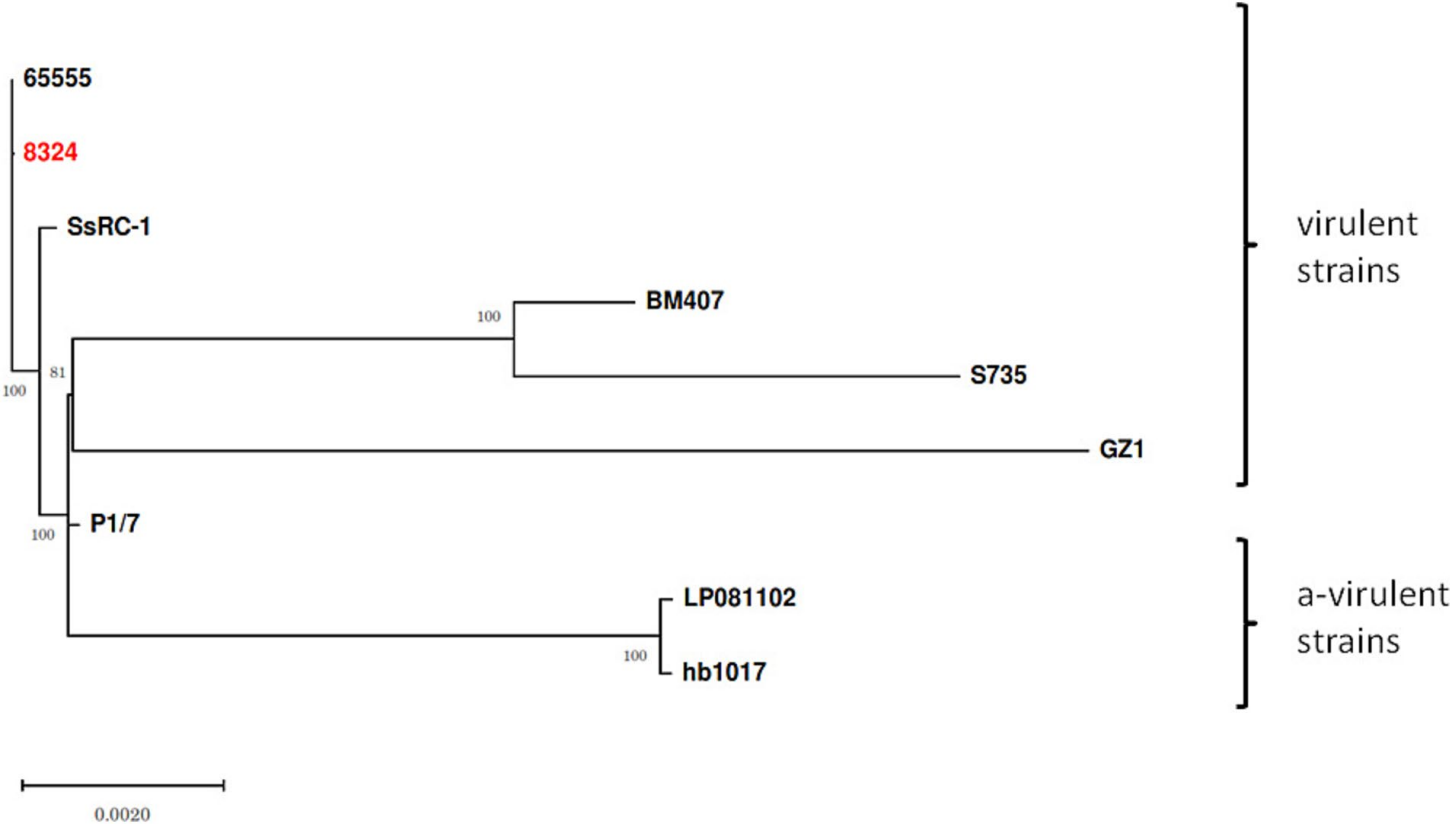
*Streptococcus suis* ST1 strains and phylogenetic relationships. The phylogenetic tree was built including virulent (65555, 8324, SsRC-1, BM407, S735, GZ1, and P1/7) and avirulent (LP081102 and hb1017) strains to assess phylogenetic distances and relationships. The strain isolated in the present study is highlighted in red, while bootstrap values are shown at branch points.

Finally, a phylogenetic tree constructed on the core genome SNP to examine the relationship among ST1 isolates from different backgrounds (Figure 3) showed a clear separation between virulent (65555, 8324, SsRC-1, BM407, S735, GZ1, and P1/7) and avirulent (LP081102 and hb1017) strains, confirming that they are phylogenetically distinct. Table 2 reports the details of the SNPs. Furthermore, high homology was displayed between the present study strain and the sequence of strain 65555, previously isolated from a case of meningitis in an adult man from the same geographical area, and they both were highly similar to SsRC-1 and P1/7, which were isolated in the same area a few years earlier from a human and a pig case, respectively.

**Table 2:** Analysis of the SNP between the isolates.

	P1/7	BM407	GZ1	SsRC-1	LP081102	Strain 65555	S735	Strain 8324	hb1017
P1/7	0	852	1519	82	910	98	1331	98	908
BM407	852	0	2323	902	1726	918	835	918	1724
GZ1	1519	2323	0	1567	2395	1583	2748	1583	2393
SsRC-1	82	902	1567	0	960	64	1381	64	958
LP081102	910	1726	2395	960	0	976	2205	976	32
Strain 65555	98	918	1583	64	976	0	1397	0	974
S735	1331	835	2748	1381	2205	1397	0	1397	2203
Strain 8324	98	918	1583	64	976	0	1397	0	974
hb1017	908	1724	2393	958	32	974	2203	974	0

## 4 Discussion

*S. suis* is reported to be part of the normal microbiota of the upper respiratory tract, gut, and genital tract of pigs, reaching up to 100% of the colonization rate in this species. Nevertheless, in some cases, the expression of a wide variety of virulence factors that facilitate the adhesion to the epithelial cells, the dissemination into the host bloodstream, neuro-invasion, as well as immune-system escape (Tram et al., 2021; Uruén et al., 2022), can be responsible for the disease. Therefore, *S. suis* can be considered a pathobiont (Vötsch et al., 2018). Indeed, the bacterium in 4–12 week-of-age piglets can easily reach the bloodstream and nervous system, leading to septicemia and meningitis, respectively, and death (Uruén et al., 2022). Furthermore, *S. suis* constitutes a growing concern for its zoonotic role, as an increasing incidence of human infections is reported worldwide. Several studies have reported *S. suis*-related meningitis cases in adults, with the main risk factor being contact with pigs or the consumption of contaminated raw pork meat (Tram et al., 2021; Tall et al., 2016). However, the most worrying aspect of this bacterium is the widespread findings of antimicrobial-resistant (AMR) strains. This phenomenon dates back to the 1980s when the first AMR strains were identified. Since then, detections of antimicrobial-resistant strains have dramatically increased over time. In Europe, the antibiotics to which the highest resistance has been detected are macrolides, lincosamides, and tetracyclines (Uruén et al., 2022), a fact that has been attributed to the excessive use of antibiotics in the swine industry. Indeed, some antimicrobials, such as tylosin, have been widely used as growth promoters, while lincosamides and tetracyclines have been largely used for their broad spectrum. Therefore, the selection pressure has favored the emergence of *S. suis* strains harboring antimicrobial resistance determinants (Uruén et al., 2022). In this context, several AMR genes have been identified in the *S. suis* genome, and the use of whole-genome sequencing (WGS) can be a useful tool to monitor the AMR genes (World Health Organization, 2020), which may be helpful in the approach of a specific therapy based on the resistance profile (Cordero and Ashley, 2012). Nevertheless, to date, WGS studies of *S. suis* are scarce (Prüfer et al., 2019). Therefore, the purpose of the present study was to characterize the whole genome of a *Streptococcus suis* strain isolated from a newborn with meningitis, in order to better understand the molecular mechanisms responsible for the virulence of the strain, its ability to cause host infection in the host, and to evaluate its antimicrobial resistance profile. Based on the presence of several genes involved in cellular adhesion, immune evasion mechanisms, and toxin production, associated with the presence of specific pili, the isolated *S. suis* was classified as a serotype 2 ST1 high virulent strain (Guo et al., 2021). Indeed, serotype 2 is considered to be the most pathogenic *S. suis*, as it is strongly associated with septicemia, meningitis, and arthritis both in humans and animals (Hughes et al., 2009; Guo et al., 2021). Furthermore, the isolated strain showed two genes responsible for macrolide and tetracycline resistance, *ermB* and *tet(W)*, respectively. Moreover, the high homology between the present study strain and previously characterized strains isolated from humans and animals may suggest the presence/persistence of a specific serotype in a determined area, which is characterized by a high density of pork farms, even in home-reared conditions. As *S. suis* can survive in the dust, fertilizer, and pig carcasses for days or even weeks under suitable conditions, the surrounding environment of hoggeries and slaughterhouses may represent a source of human infection (Gottschalk et al., 2010).

Nevertheless, in the present study, the epidemiological survey found no close family members working in *S. suis* infection risk-associated activities. Indeed, pig-related occupations are described as the main risk factor for human contagion, but exposure is not always reported (Hughes et al., 2009). In our case, the bacterium was detected in the upper respiratory airways of all family members, which may represent a potential risk of transmission and spread of the pathogen. Furthermore, the infant was born preterm in the 36th week of pregnancy. Preterm birth is reported to occur before the 37th week of gestation and is closely associated with the microbiological composition of the mucosa of the reproductive tract of a pregnant woman. Indeed, a study evaluating the microbiota pathways of the vaginal mucosa of pregnant women evidenced the presence of *S. suis*, in addition to *Gardnerella vaginalis*, *Lactobacillus crispatus*, and *Fannyhessea vaginae*, was more frequent in preterm birth and neonatal septicemia compared to the control group with normal newborns (Yang et al., 2023). Cases of neonatal sepsis caused by group B Streptococcus at the early onset before the 7th day of birth and late onset between the 7th and 89th day of birth are known (Fister et al., 2022). In our case, it can be hypothesized that a case of late meningitis with transmission from the mother during child delivery may occur also for *Streptococcus suis*. It can be assumed that the mother’s infection occurred because of her habit of consuming a traditional product of raw pig origin, known as “Nduja,” consisting of pork offal and tripe or jowl bacon and bacon; therefore, it cannot be excluded that the mother was colonized by *S. suis* through the ingestion of contaminated food and vertically transmitted the bacterium to the newborn (Figure 1).

## 5 Conclusion

Although *S. suis* is mainly reported in adults with risk factors associated with close contact with pigs or the consumption of raw or poorly cooked swine meat (Hughes et al., 2009), our results highlight the need for early research/diagnosis in pregnant women, especially in areas with a high density of pig farms, where there is a high rate of handling or consumption of raw meat, in order to reduce newborn infections. Furthermore, it is important to underline the importance of the evaluation of the AMR profile of *S. suis* strains in order to assess the potential emergence of further resistance and allow for targeted therapy.

## Data Availability

The data that support the findings of this study are openly available in National Library of Medicine at https://www.ncbi.nlm.nih.gov/, reference number CP141904.

## Footnotes

^1^ https://www.bioinformatics.babraham.ac.uk/projects/fastqc/

^2^ https://ccb.jhu.edu/software/kraken2/

^3^ https://pubmlst.org/sagalactiae/

^4^ https://github.com/aquacen/serotype_Sagalactiae

^5^ https://cge.cbs.dtu.dk/services/ResFinder/
